# Development of a target product profile for a point-of-care cardiometabolic device

**DOI:** 10.1186/s12872-021-02298-7

**Published:** 2021-10-09

**Authors:** Beatrice Vetter, David Beran, Philippa Boulle, Arlene Chua, Roberto de la Tour, Lucy Hattingh, Pablo Perel, Gojka Roglic, Rangarajan Sampath, Michael Woodman, Sigiriya Aebischer Perone

**Affiliations:** 1 FIND, 9 Chemin des Mines, 1202 Geneva, Switzerland; 2 Division of Tropical and Humanitarian Medicine, University of Geneva and Geneva University Hospitals (UNIGE/HUG), Geneva, Switzerland; 3 Médecins Sans Frontières (MSF), Geneva, Switzerland; 4 LH Consulting, Berkeley, CA USA; 5 Centre for Global Chronic Conditions, London School of Hygiene and Tropical Medicine (LSHTM), London, UK; 6 Department for Noncommunicable Diseases, World Health Organization (WHO), Geneva, Switzerland; 7 United Nations High Commissioner for Refugees (UNHCR), Geneva, Switzerland; 8 International Committee of the Red Cross (ICRC), Geneva, Switzerland

**Keywords:** Cardiovascular disease, Diabetes, In vitro diagnostics, Medical device, Low- and middle-income country, Primary care, Multi-parameter device

## Abstract

**Introduction:**

Multi-parameter diagnostic devices can simplify cardiometabolic disease diagnosis. However, existing devices may not be suitable for use in low-resource settings, where the burden of non-communicable diseases is high. Here we describe the development of a target product profile (TPP) for a point-of-care multi-parameter device for detection of biomarkers for cardiovascular disease and metabolic disorders, including diabetes, in primary care settings in low- and middle-income countries (LMICs).

**Methods:**

A draft TPP developed by an expert group was reviewed through an online survey and semi-structured expert interviews to identify device characteristics requiring refinement. The draft TPP included 41 characteristics with minimal and optimal requirements; characteristics with an agreement level for either requirement of ≤ 85% in either the survey or among interviewees were further discussed by the expert group and amended as appropriate.

**Results:**

Twenty people responded to the online survey and 18 experts participated in the interviews. Twenty-two characteristics had an agreement level of ≤ 85% in either the online survey or interviews. The final TPP defines the device as intended to be used for basic diagnosis and management of cardiometabolic disorders (lipids, glucose, HbA1c, and creatinine) as minimal requirement, and offering an expanded test menu for wider cardiometabolic disease management as optimal requirement. To be suitable, the device should be intended for level 1 healthcare settings or lower, used by minimally trained healthcare workers and allow testing using self-contained cartridges or strips without the need for additional reagents. Throughput should be one sample at a time in a single or multi-analyte cartridge, or optimally enable testing of several samples and analytes in parallel with random access.

**Conclusion:**

This TPP will inform developers of cardiometabolic multi-parameter devices for LMIC settings, and will support decision makers in the evaluation of existing and future devices.

**Supplementary Information:**

The online version contains supplementary material available at 10.1186/s12872-021-02298-7.

## Background

Although non-communicable diseases (NCDs) are often thought to be a problem of high income countries, a large proportion of the burden of NCDs is borne by low- and middle-income countries (LMICs), with 78% of all NCD-related deaths and 85% of premature NCD-related deaths in people aged between 30 and 69 years occurring in these settings [1, 2]. Cardiovascular diseases (CVDs) and metabolic disorders represent a large proportion of the NCD burden in LMICs [3, 4], with stroke, ischaemic heart disease, diabetes and chronic kidney disease commonly appearing in the top ten causes of life years lost due to premature mortality [5]. Unlike high-income countries, many LMICs lack the healthcare resources to tackle this increasing burden [2, 3]. Primary healthcare, with its emphasis on promoting health and preventing disease, is the most effective way to reduce premature mortality from NCDs [6, 7], but many primary healthcare facilities in LMICs lack the laboratory capacity required for diagnosis and monitoring of these conditions [8]. As such, the World Health Organization (WHO) Global Action Plan for NCDs recommends improvement of diagnostic services for the four NCDs with the highest contribution to morbidity and mortality, including CVDs and diabetes, as well as promotion of development and equitable dissemination of affordable, effective and quality diagnostics for these NCDs [9].

Prevention, diagnosis and management of CVDs and diabetes is achieved through the monitoring of various laboratory parameters such as lipids as a risk factor for atherosclerosis, blood glucose for diabetes, serum creatinine for chronic kidney disease, and liver enzymes for liver disease. Based on the results of laboratory tests, best treatment options are chosen and dosages are adapted. Multi-parameter diagnostic devices, which can test for multiple analytes either simultaneously or sequentially from a single sample, hold the potential to streamline and simplify cardiometabolic disease diagnosis and management [10]. However, while several multi-parameter devices for CVDs already exist, they may not be suitable for use in LMICs due to resource requirements (e.g. power, storage), the need for trained users, and environmental operating conditions [11].

There is a demonstrated need to develop and adopt affordable and effective point-of-care (POC) diagnostic tools that are suitable for use in low-resource primary care settings, to improve diagnosis and management of cardiometabolic disease [12]. Here, we describe the development of a target product profile (TPP) for a POC multi-parameter device to measure cardiometabolic biomarkers in LMIC primary care. The TPP aims to define the minimal and optimal requirements for a device suitable for use in this setting.

## Methods

The TPP was developed in three stages: (1) preparation of a draft TPP for diagnosis of cardiometabolic diseases by an expert group; (2) consensus building through online survey and expert interviews to identify device characteristics for further refinement, and (3) TPP finalization by the expert group.

## Draft TPP preparation

Baseline TPP requirements were taken from a previously developed TPP (version 0) developed by WHO, FIND, and MSF, which described desired characteristics of a multi-parameter POC polymerase chain reaction (PCR) machine to diagnose infection with pathogens causing febrile illness [13]. Version 0 included 41 characteristics relating to the scope of the device, the instrument, and the assay cartridge, each with minimal and optimal requirements. Version 0 had been fully vetted using a Delphi-like process, involving a stakeholder survey of 52 experts followed by a TPP working group discussion to address characteristics with low agreement; the process was then repeated, and the revised draft was put forward for a month of public consultation on the WHO and FIND websites.

For the cardiometabolic device TPP development, an expert group was convened to adapt the previously developed TPP (version 0) to create a draft TPP for diagnosis of cardiometabolic diseases (version 1). Members were selected from healthcare organizations or academic centres with an interest in improving health in low-income settings, and represented organizations with relevant expertise in NCDs, diagnostics or laboratory work. Areas of expertise for each participating expert group member are shown in Table 1. The expert group meeting took place on 12th December 2019 in Geneva, Switzerland. During the meeting, version 0 of the TPP was adapted to the context of cardiometabolic non-communicable diseases and their management at the primary healthcare level, resulting in amendments to the intended use and target use setting characteristics. Device and assay cartridge configurations were also adapted to reflect the detection of biochemical parameters, rather than the PCR nucleic acid amplification techniques used for infectious disease. This included amendments to characteristics relating to device design, type of parameters, technical aspects for measurement, turnaround times and test results. List prices were also adjusted. The resulting TPP (version 1) is shown in Additional file 1.

**Table 1:** Expert group expertise and experience

Expert affiliation	Expertise/role	Highest degree
FIND	Scientific officer, non-communicable diseases lead	PhD
FIND	Chief Scientific Officer, technical product development	PhD
ICRC	Specialist in general internal medicine, humanitarian conflict physician	MD
LH Consulting	Medical diagnostics business consultant	MBA
LSHTM	Cardiovascular clinical epidemiologist	MD, MSc
MSF	Non-communicable diseases advisor and working group leader, physician and international public health specialist	MPH
MSF	Diagnostics network leader, physician and public health specialist	MD, MSc
MSF	Laboratory advisor	PhD
UNHCR	Senior Public Health Officer, humanitarian physician	MPH
UNIGE/HUG	Lecturer and researcher, public health specialist in NCDs, diabetes and health systems	PhD
WHO	Medical Officer, physician and epidemiologist	MSc

*FIND*, the global alliance for diagnostics, *ICRC* International Committee of the Red Cross, *LH* Lucy Hattingh, *LSHTM* London School of Hygiene and Tropical Medicine, *MSF* Médecins Sans Frontières, *UNHCR* United Nations High Commissioner for Refugees, *UNIGE/HUG* University of Geneva/Hôpitaux Universitaires Genève, *WHO* World Health Organization

### Consensus building

A two-step method was employed to facilitate consensus building for the TPP. Firstly, the draft TPP (version 1) was reviewed through an online survey. Secondly, semi-structured stakeholder interviews were performed in order to obtain additional feedback on relevant or controversial areas.

The online survey was created using Alchemer, formerly Survey Gizmo, software. A link to the online survey was posted on the FIND LinkedIn account (> 10,000 followers) and Twitter account (> 7000 followers). Members of the expert group also distributed the link amongst their respective networks. The survey was open from 14 February 2020 to 30 April 2020. Survey respondents were asked to rate their level of agreement with each of the 41 minimal and 41 optimal requirements in the draft TPP (version 1) using a 5-point Likert scale [14]. Percentage agreement was determined by the number of respondents with a ‘mostly agree’ or ‘fully agree’ rating (score of 4 or 5), and disagreement with a criterion based on a rating of ‘fully disagree’, ‘mostly disagree’ or ‘neither agree nor disagree’ (scores from 1 to 3), which required a comment from the survey respondent to explain their reasons for disagreement. Respondents could provide additional comments to accompany scores of 4 or 5 if desired, but this was not mandatory.

Clinicians, laboratory experts and procurers of POC cardiometabolic devices were targeted for the semi-structured stakeholder interviews. Eligible participants were those who influence or make key decisions on purchase or use of POC cardiometabolic devices at the primary place of usage, and who self-rate as having at least fair or very familiar knowledge with these devices. An expert search agency was employed to identify eligible respondents using a screening questionnaire, aiming to match the number of interviewees to the number of survey respondents as closely as possible. Interviewees were recruited and interviews were conducted between June and July 2020. Interviews were performed by video call and were aided by a semi-structured discussion guide (Additional file 2). Calls were recorded with the respondents’ permission and analysis was conducted on artificial intelligence-generated transcripts. Notes were taken wherever permission for recording was not provided. Interviews were performed by two employees of IQVIA Inc. (Durham, NC, USA). The interviewers were experienced in qualitative and quantitative research in the healthcare industry, and held social science qualifications (Bachelor of Arts in Social Science and Master of Science in Social Research Methods, respectively). The interviewers did not know any of the persons interviewed prior to this study.

Interviewees were catergorized into device users, purchase decision makers or both, based on their feedback from the screening questionnaire. Interviewees were shown 29 of the 41 device characteristics with minimal and optimal requirements from version 1 of the TPP, relevant to their area of expertise, and asked to identify the top ten characteristics that were most important. The order in which the characteristics were shown was rotated for each interviewee to reduce order bias. Eleven characteristics were not included in the interviews as their requirements were deemed less likely to require adaptation due to the stringent baseline definition (data protection, manufacturing quality, regulatory approval, performance criteria, sample volume, memory). The interviews were qualitative in nature; however, characteristics that were mentioned by more than half of the interviewees were also quantified. Quantitatively rated characteristics were scored as a percentage, with the number of interviewees who identified a characteristic as being important to them as the denominator and the number who agreed with the minimal or optimal requirement as the numerator. Separate to the TPP development, interviewees were also asked to rank the characteristics that they identified in the order of most importance.

### TPP finalization

To finalize the TPP, the expert group reconvened to discuss the TPP characteristics with an agreement level of ≤ 85% for either the minimal or optimal requirement based on either the survey or the interview results. The meeting was virtual and took place on 4 September 2020 (two experts were excused). The highest priority characteristics were discussed in detail until agreement was reached, while lower priority characteristics were voted on by the expert group to achieve a consensus. The majority (≥ 50%) of respondents who voted on each characteristic needed to vote in favour of an amendment in order for it to be made.

### Ethics and consent

As this research did not include human or animal subjects, no ethical or licensing committee approvals or informed consent was required. There are no specific regulations or guidelines for the development of TPPs, however, the methodology used in this study was consistent with protocols for previous TPPs developed by FIND and/or WHO.

## Results

### Online survey and semi-structured interviews

Of 65 people who accessed the online survey, 20 responded, of whom 13 provided complete responses. Respondents were from 15 countries, and the majority were employees of or consultants for non-governmental organizations (n = 7) or medical doctors (n = 6) (Table 2). For the interviews, eighteen experts agreed to participate. The majority were from South Africa (n = 6) and India (n = 5), and most were clinical experts (Table 2).

**Table 2:** Characteristics of online survey respondents and interviewees

Characteristic	Number
*Survey respondents (N = 20)*	
Country	
Germany	1
India	2
Iraq	2
Italy	2
Switzerland	2
United Kingdom	2
Canada	1
Egypt	1
Lebanon	1
Malawi	1
Netherlands	1
Nigeria	1
Uganda	1
Ukraine	1
United States	1
Profession	
Employee/consultant for NGO*	7
Medical doctor	6
IVD diagnostics industry personnel	1
Biomedical Engineer	1
Consultant	1
Epidemiologist	1
Laboratory expert	1
Nurse	1
Public Health	1
*Interviewees (N* = *18)*	
Country	
South Africa	6
India	5
Peru	2
Uganda	2
Bangladesh	1
Brazil	1
Tanzania	1
Primary role	
Clinical	12
Laboratory	4
Procurement	2

*IVD* in vitro diagnostics, *NGO* non-governmental organization

*National or international

**Fig. 1:**
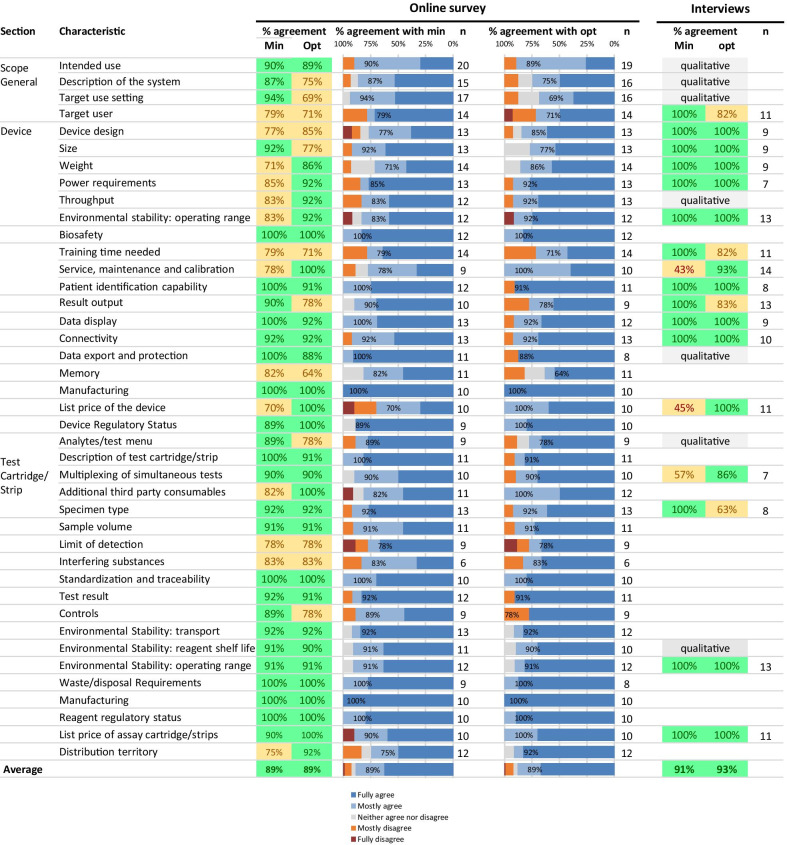
Results from the online survey and semi-structured interviews

Results from the survey and interviews are shown in Fig. 1. In the online survey, of the 41 minimal requirements, 14 had an agreement level of ≤ 85%. Minimal requirements with the lowest agreement were list price of the device (70%), weight of the device (71%), and distribution territory (75%). Of the 41 optimal requirements, 12 had an agreement level of ≤ 85%. Optimal requirements with the lowest agreement were device memory (64%), target use setting (69%), target user (71%) and training time needed (71%). In the interviews, minimal requirements with a quantitative assessment that had an agreement level of ≤ 85% were service, maintenance and calibration (43%), list price of the device (45%) and multiplexing of simultaneous tests (57%). Optimal requirements with a quantitative assessment that had an agreement level of ≤ 85% were target user (82%), training time needed (82%), result output (83%), and specimen type (63%). Only two characteristics that had an agreement level of > 85% for either requirement in the online survey had an agreement level of ≤ 85% in the interviews (multiplexing of simultaneous tests and specimen type).

### TPP finalization

In addition to the 22 characteristics with low disagreement in the survey and interviews, the intended use characteristic was also discussed during the expert meeting. While there was a high level of agreement on this characteristic among the survey respondents and interviewees, the intended use is directly linked to the test menu, where agreement was lower. Moreover, survey respondents made a range of comments on this question, so the experts felt it necessary to revisit discussions to confirm the existing description was appropriate. The intended use characteristic was modified to clarify that the scope of the TPP includes management of people with high cardiovascular risk, as well as diagnosis and management of people with cardiometabolic disorders.

While some survey respondents and interviewees disagreed with the optimal target use setting requirement (level 0 health facility without equipped laboratory, electricity with frequent surges and/or outages, no climate control, dusty environment; includes mobile testing facilities; medical staff onsite), experts decided to retain this wording, since optimal requirements always define an ideal device. The minimal target user characteristic was reworded to clarify that while general laboratory training was not required for users of the POC device, specific training for the multi-parameter device would need to be provided; for the optimal requirement, users should be capable of applying this specific device training. This characteristic was renamed ‘target operator’.

In version 1 of the TPP, the minimal requirement for the device design characteristic allowed for the test menu to be covered by multiple instruments. However, the survey respondents felt that this may not be cost effective and would introduce redundancy. The expert group therefore amended the minimal requirement to require a single device to cover the minimal test menu, and the possibility for several devices to be connected to run the same tests in parallel was moved to the optimal requirement. Additionally, based on survey feedback that hand-held devices have limited added value over small tabletop devices for use in primary care settings, and may not be preferred due to greater potential for hand-held devices to be lost, dropped or misplaced, the optimal requirement for the size of the device was amended to require the device to be portable rather than hand-held. Weight requirements were also amended from ≤ 15 kg to  ≤ 10 kg for the table-top device (minimal) and from ≤ 1 kg to ≤ 2 kg for the portable device (optimal).

Survey respondents were concerned that operational characteristics of the device were not sufficient for countries in which average summer temperatures are higher than 35°C. The temperature ranges were subsequently increased; a requirement for the device to be water splash proof was also added. Survey respondents and interviewees felt that the training times for users in both the minimal and optimal requirements were too optimistic; however, they believed that this had been interpreted in the context of patient management rather than device operation, and so the wording ‘to operate the device’ was added to both requirements. Based on interviewee feedback, minimal maintenance requirements were changed from daily to weekly. Survey respondents commented that regional variation in result output should be discouraged; this was therefore removed from the optimal requirement.

There was considerable feedback from survey respondents and interviewees regarding the list price of the device, with many commenting that the minimal cost of 5,000 USD would be an extremely high capital investment for LMIC healthcare centres. Finding the optimal trade-off point between affordability and device sophistication is challenging. However, recurrent costs of the tests may be more important than device cost, as diagnostic devices are often provided free of charge or for a small service charge provided that a minimum number of tests are purchased within a defined period of time. After much debate, the minimal requirement for list price of the device was lowered to 1,500 USD, noting that a higher price might be acceptable under specific circumstances such as reagent lease or rental agreements.

With regards to the test menu characteristic, respondents though the minimal test menu should include explicit result outputs for total cholesterol (TC) and high-density lipoprotein (HDL) to calculate low-density lipoprotein (LDL), rather than limiting the output to calculated LDL, without specific result output for TC and HDL to the user. The minimal requirement was adapted accordingly. Survey respondents commented that troponin may not be a relevant parameter for settings of intended use of the device, however, it was decided to keep this parameter for optimal requirement not to exclude a use case for the device, even if unlikely. Interviewees thought that testing of one analyte at a time would be too time consuming, however, experts noted that the device should allow use of single cartridges to permit individual tests to be conducted at different frequencies and prevent waste. The minimal requirement for multiplexing of simultaneous tests was therefore changed to ‘Testing of one analyte at a time in single or multi-analyte panel cartridge’. Some respondents commented that more interfering substances should be included, therefore both requirements were amended to state that interference testing should follow Clinical and Laboratory Standards Institute (CSLI) EP37 guidance on substances and threshold levels [15], as these are internationally recognized standards.

It was noted that different analytes may require different sample types, thus the minimal requirement for fingerstick whole blood may be too restrictive. To allow more flexibility around specimen types, the minimal requirement was amended to allow for use of plasma, serum or urine samples, in addition to whole blood, with a limitation of one specimen type per cartridge or strip. The optimal requirement was amended to allow for different specimen types per cartridge or strip. The sample volume requirement was subsequently changed to prescribe a specific volume for fingerstick whole blood only, as the most difficult specimen type for which to collect sufficient volume.

Following feedback from online survey respondents that the test should not be limited to certain regions, it was agreed to change the minimal requirement for distribution territory to ‘worldwide’, and the optimal requirement to ‘same as minimal’.

Other minor amendments included renaming of the ‘limit of detection’ and ‘description of the system’ characteristics to ‘accuracy’ and ‘description of the device’, and clarifying that clinical decision- making based on test results should be performed by clinicians/medical staff. Overall, minimal and/or optimal requirements were adjusted for 18 of the 23 characteristics discussed. The final TPP is shown in Table 3.

**Table 3:** Finalized TPP for a multi-parameter cardiometabolic POC device

#	Characteristic	Min/Opt	Requirements
*General*			
1	Intended use	Minimal	Intended for basic screening, diagnosis and management of cardiometabolic disorders (e.g. hyperlipidaemia, diabetes and renal function) and also managing people at high cardiovascular risk; excluding neonates
1a		Optimal	Same as minimal, plus offering an expanded test menu to address a wider range of cardiometabolic disorders (e.g. liver function, acute cardiac care); including neonates
2	Description of device	Minimal	Benchtop (or hand-held) instrument designed for use in combination with self-contained, disposable assay cartridge(s) or strips containing all required reagents to execute a test from sample to result
2a		Optimal	Same as minimal
3	Target use setting	Minimal	Level 1 healthcare facility (primary care) defined as having a rudimentary equipped laboratory, water, electricity with intermittent surges and/or outages, limited climate control, dusty environment; medical staff onsite
3a		Optimal	Level 0 healthcare facility without equipped laboratory, electricity with frequent surges and/or outages, no climate control, dusty environment; includes mobile testing facilities; medical staff onsite
4	Target operator	Minimal	Minimally skilled healthcare worker e.g. with basic laboratory training (device-specific training provided)
4a		Optimal	Healthcare worker without specific laboratory training (capable of applying device-specific training)
*Device*			
5	Device design	Minimal	Device with single port capable of interfacing with one cartridge design or strip
5a		Optimal	Device with several ports capable of interfacing with one or more cartridge designs or strips for simultaneous, independent detection of multiple analytes; possibility for modular connectivity of several devices
6	Size	Minimal	Small, table-top device (no larger than 50 × 70 × 50 cm)
6a		Optimal	Smaller than minimal and portable
7	Weight	Minimal	≤ 10 kg
7a		Optimal	≤ 2 kg
8	Power requirements	Minimal	Local 110–220 V AC mains power, plus uninterruptible power supply (UPS) to complete current cycle; UPS and circuit protector must be integrated within the system
8a		Optimal	Same as minimal, with rechargeable battery back-up (8-h operation) or single-use battery (for hand-held)
9	Throughput	Minimal	Throughput processing of one sample at a time; minimum of 10 samples per hour when individual analytes are tested or 4 samples per hour when analyte panels are tested
9a		Optimal	More than one sample at a time with random access and the ability to test different analytes simultaneously
10	Environmental Stability: operating range of the device	Minimal	Operation at 10–40 °C and up to 90% non-condensing humidity at an altitude up to 2500 m; able to function in direct sunlight; able to withstand dusty conditions; water splash proof
10a		Optimal	Operation at 5–45 °C and up to 98% non-condensing humidity at an altitude up to 3000 m; able to function in direct sunlight; able to withstand dusty conditions; water splash proof
11	Biosafety	Minimal	Closed, self-contained system with unprocessed sample transfer; easy decontamination of instrument surfaces
11a		Optimal	Same as minimal
12	Training time needed	Minimal	Below 1 day for a healthcare worker to operate the device
12a		Optimal	Below 2 h for a healthcare worker without basic laboratory training to operate the device
13	Service, maintenance and calibration	Minimal	Weekly maintenance (< 30 min, with hands on time < 10 min); mean time between failures of at least 24 months or 10,000 tests; self-check alerting operator to instrument errors or warnings; operator calibration per new lot or at set time intervals
13a		Optimal	Weekly maintenance (< 30 min, with hands on time < 10 min); mean time between failures of at least 36 months or 30,000 tests; self-check alerting operator to instrument errors or warnings; ability to be calibrated remotely or no calibration needed (factory calibrated)
14	Patient identification capability	Minimal	Manual entry of alphanumeric patient identifier via keypad, touchscreen or connected result management device (e.g. smartphone)
14a		Optimal	Same as minimal, plus bar code, radio frequency identification (RFID) or other reader
15	Result output	Minimal	Quantitative based on the analytes of detection; qualitative where this is sufficient to inform clinical decision making
15a		Optimal	Quantitative plus option of qualitative readout where that result is sufficient to inform clinical decision-making; ability to select which test results are reported to the user
16	Data display	Minimal	On-device visual readout with ability to function in various lighting conditions ranging from bright to low ambient light conditions; ability to add information (patient ID, operator ID, date, location, etc.)
16a		Optimal	Same as minimal, with option to add custom result ranges and alerts to support clinical decision-making by medical staff
17	Connectivity	Minimal	Ability to connect to a mobile network, or Wifi or use a USB for data transfer
17a		Optimal	Same as minimal, including bluetooth and bi-directional communication
18	Data export and protection	Minimal	Secured data export with end-to-end encryption connectivity to external printer; passcode-protected machine access
18a		Optimal	Same as minimal, plus scheduled/automatic data export using interoperable standards; support of any or all of the following formats: HL7, FHIR, ASTM, JSON; passcode-protected individual user access
19	Memory	Minimal	500 patient results, 100 quality control (QC) results
19a		Optimal	10,000 patient results, 20,000 QC results or unlimited data storage (cloud-based)
20	Manufacturing	Minimal	International Organization for Standardization (ISO) 13,485:2016 compliant
20a		Optimal	Same as minimal
21	List price of the device	Minimal	≤ 1,500$ (USD)
21a		Optimal	≤ 300$ (USD)
22	Device regulatory status	Minimal	Approval through at least one Stringent Regulatory Authority (http://www.stoptb.org/assets/documents/gdf/drugsupply/List_of_Countries_SRA.pdf)
22a		Optimal	Same as minimal plus CLIA-waived; WHO-PQ approval if requirements are in place
*Test cartridge/strip*			
23	Analytes/test menu	Minimal	Glucose, HbA1c, lipids (total cholesterol and HDL to calculate non-HDL cholesterol), creatinine
23a		Optimal	Same as minimal and full lipid profile (values for cholesterol, HDL, LDL and triglycerides), liver enzymes (ALT, AST, ALP, GGT, bilirubin), troponin, BNP, ACR, auto calculation of eGFR and others as required for wider cardiometabolic disease management
24	Description of test cartridge/strip	Minimal	Self-contained, disposable cartridge(s)/strips containing all required reagents, buffers or other consumables to execute a test from sample to result
24a		Optimal	Same as minimal
25	Multiplexing of simultaneous tests	Minimal	Testing of one analyte at a time in single or multi-analyte panel cartridge
25a		Optimal	Testing of several analytes in parallel, either with multi-analyte panel cartridge, or with several cartridge/strip ports; ability to measure analytes individually, as well as part of a panel
26	Additional third party consumables	Minimal	None, except for sample collection
26a		Optimal	None; manufacturer-provided kits contain all required items for sample collection and testing
27	Specimen type	Minimal	Ability to accept one specimen type per cartridge/strip (whole blood or plasma or serum or urine, depending on the parameter)
27a		Optimal	Ability to accept different specimen types per cartridge/strip (whole blood, plasma, serum, urine; non-exclusive with exception of parameter dependency on sample type)
28	Sample volume	Minimal	Minimum sample volume required to reach clinically relevant sensitivities for each test; no more than 50 μl per parameter for fingerstick whole blood (cumulative volume for panel cartridges)
28a		Optimal	Same as minimal
29	Accuracy	Minimal	Equivalent to state of the art reference assays for the same target analytes; where applicable, clinically relevant LODs are to be met; for troponin, rule-out of myocardial infarction according to ACC/AHA guidelines
29a		Optimal	Same as minimal; for troponin: rule-out of myocardial infarction according to ESC 2018 guidelines
30	Interfering substances	Minimal	Interference testing should follow CLSI EP37 list of recommended substances
30a		Optimal	Same as minimal
31	Standardization and traceability	Minimal	Test should be standardized based on established methods (e.g. isotope dilution mass spectrometry, ID-MS) and traceable to internationally recognised reference materials (where available)
31a		Optimal	Same as minimal
32	Test result	Minimal	Quantitative result based on the analytes of detection. Qualitative result available to clinician where that result is sufficient to inform clinical decision-making
32a		Optimal	Same as minimal
33	Controls	Minimal	External positive and negative controls to be run with each new lot and every week
33a		Optimal	External positive and negative controls to be run with each new lot and every month
34	Environmental stability: transport	Minimal	No cold chain required; should be able to tolerate stress during transport (cycles of temperature of 30 to 50 °C) without affecting the labelled expiry date
34a		Optimal	Same as minimal
35	Environmental Stability: Reagent shelf life	Minimal	18 months at 2–35 °C (including 3 months at 40 °C); 90% relative humidity
35a		Optimal	24 months at 2–40 °C; up to 98% relative humidity
36	Environmental Stability: Operating range	Minimal	10–40 °C; 90% relative humidity
36a		Optimal	5–45 °C; 98% relative humidity
37	Waste/disposal Requirements	Minimal	No components that are classified with a GHS[1] classification—H(2) that would require waste disposal with high temperature incinerator (or more than a De Monfort type incinerator)
37a		Optimal	Same as minimal
38	Manufacturing	Minimal	International Organization for Standardization (ISO) 13,485:2016 compliant
38a		Optimal	Same as minimal
39	Reagent regulatory status	Minimal	Approval through at least one Stringent Regulatory Authority (http://www.stoptb.org/assets/documents/gdf/drugsupply/List_of_Countries_SRA.pdf)
39a		Optimal	Same as minimal plus CLIA-waived; WHO-PQ approval if requirements are in place
40	List price of assay cartridge/strips	Minimal	Strips: ≤ 1$ (USD); cartridges: ≤ 3$ (USD) per analyte (individual or as part of a panel)
40a		Optimal	Strips: ≤ 0.5$ (USD); cartridges: ≤ 1$ (USD) per analyte (individual or as part of a panel)
41	Distribution territory	Minimal	Worldwide
41a		Optimal	Same as minimal

*ACC* American College of Cardiology, *ACR* albumin-to-creatinine ratio, *AHA* American Heart Association, *ALT* alanine aminotransferase, *ALP* alkaline phosphatase, *AST* aspartate aminotransferase, *ASTM* American Society for Testing and Materials, *BNP* brain natriuretic peptide, *CLIA* Clinical laboratory improvement amendments, *CSLI* Clinical and Laboratory Standards Institute, *ESC* European Society of Cardiology, *FHIR* fast healthcare interoperability resources, *eGFR* estimated glomerular filtration rate, *GGT* gamma-glutamyl transferase, *GHS* globally harmonized system of classification and labelling of chemicals, *HbA1c* glycated haemoglobin, *HDL* high-density lipoprotein, *HL7* health level 7, *ID* identification, *ID-MS* isotope dilution mass spectrometry, *ISO* International Organization for Standardization, *JSON* JavaScript object notation, *LDL* low-density lipoprotein, *LOD* limit of detection, *RFID* radio frequency identification, *QC* quality control, *UPS* uninterruptible power supply, *USB* Universal Serial Bus, *USD* United States dollars, *WHO-PQ* World Health Organization prequalification

### Ranking of device characteristics

**Fig. 2:**
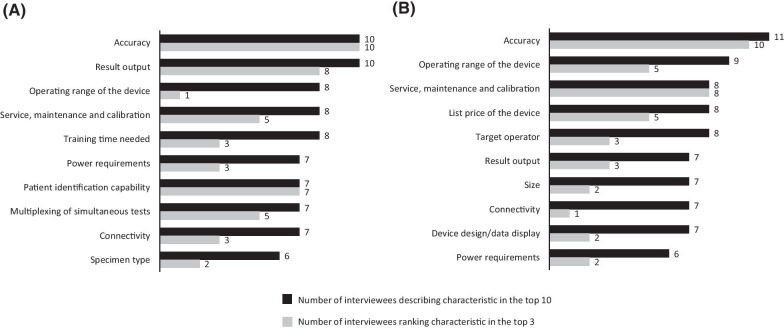
Most important device characteristics for: **a** clinicians and users (N = 13); **b** purchase decision makers (N = 12)

Device characteristic rankings from interviewees are shown in Fig. 2. For interviewees who were clinicians or potential users of the device (n = 13), the characteristics most commonly rated as important were accuracy (previously limit of detection), result output and environmental stability—operating range. The characteristics most rated among the top three most important were accuracy, result output and patient identification capability. For interviewees who were purchase decision makers (n = 12), the most commonly identified characteristics were accuracy, environmental stability—operating range, service, maintenance and calibration, and list price of the device. These were also the characteristics most commonly ranked in the top three.

## Discussion

This TPP defines the minimal and optimal requirements for a multi-parameter cardiometabolic POC device to be used in primary care settings in LMICs. The TPP aims to encourage the development of devices for the diagnosis and management of cardiovascular diseases and metabolic disorders, conditions that are becoming an increasing burden in low-resource countries. Additionally, the TPP may be used to assess existing multi-parameter devices to determine how well they might meet needs in LMIC settings [11]. The TPP is intended to be a ‘living document’, with requirements to be regularly reviewed and adapted to accommodate evolving needs and technologies.

While this TPP will inform developers and manufacturers on the key capabilities of a device for use in LMICs, we acknowledge that there is no ‘one size fits all’ approach to diagnosis and management of cardiometabolic disease and risk factors in these settings. Regional features such as target population, availability of trained specialists and on-site expertise, accessibility of related services, and purchase decision-maker requirements, will influence the exact needs of each country. The TPP was designed for primary care settings; however, primary care facilities can vary widely across regions, from basic temporary or mobile facilities in humanitarian settings to permanent centres with access to laboratory facilities, electricity and trained doctors and nurses. The ideal device would be usable across all primary care settings. Nevertheless, in recognition of the challenges involved in developing devices for use in facilities with limited resources, the minimal requirement is for level 1 healthcare facilities.

The test menu was designed to address the key cardiometabolic diseases in LMICs. Glucose and HbA1c testing for diagnosis and management of diabetes, non-HDL cholesterol testing for atherosclerotic conditions, and creatinine for kidney disease were considered the minimal requirements for the device to be of value. Optimally, the device would also allow measurement of liver enzymes, troponin and brain natriuretic peptide for myocardial infarction and heart failure, and glomerular filtration rate for kidney function. Other common cardiometabolic markers were discussed, including urea, albumin, blood ketones and thyroid-stimulating hormone. However, to ensure that the TPP requirements were not overly restrictive, it was decided to limit the optimal requirements to the analytes described above. Indeed, some survey respondents felt that there may already be too many analytes for a POC device.

Feedback from the expert interviews suggests that accuracy of the device will be the primary consideration for both clinicians and purchase decision-makers. As the results will be used to inform clinical decision making, incorrect results could lead to adverse patient outcomes, thus quantitively accurate measurements are likely to be non-negotiable requirements. Additionally, both clinicians and purchase decision makers emphasized the importance of environmental stability, especially for level 0 healthcare settings. Clinicians also rated patient identification capability as highly important, in order to allow linking of test results with other patient parameters, as well as easy-to-interpret result outputs. Purchase decision makers identified service and maintenance as key characteristics, since less frequent maintenance can lead to cost savings.

The TPP was developed using a robust multi-step process—a standard approach for the generation of such documents [16–18]. However, while TPP development commonly includes a second round of online surveying, this was not deemed necessary for this TPP, since the original TPP had already been fully vetted through both a survey and stakeholder interviews, and the first round had generated a strong body of evidence for the NCD-specific characteristics and requirements. Additionally, we used an agreement level of ≤ 85% to identify requirements for further discussion, which is more stringent than the 75% used in similar TPP development processes [16–18]. While a large proportion of survey respondents from the original TPP (version 0) were clinicians or laboratory experts, they were not necessarily the final users of such devices, which is a frequent limitation of TPPs and has the potential to influence requirements. We aimed to mitigate potential influence of this limitation in the TPP by including interviews with clinicians and laboratory experts with experience in the use of POC cardiometabolic devices. However, we cannot exclude that a degree of such influence persisted from the original TPP, as well as the online survey respondents of this TPP.

Our methodology has some limitations, including the possibility for bias. For example, the sequence in which the characteristics were presented may have led to disproportionate importance being placed on certain requirements, and the representation of the qualifications of the survey respondents may have resulted in responses being over- or under. The survey design also had the potential to encourage agreement as the quickest route to completion. The survey period coincided with the beginning of the COVID-19 pandemic, which is likely partially responsible for the low response rate, as those who would have responded under normal circumstances may have had other priorities. Additionally, as a large proportion of FIND’s work is related to infectious diseases, it is possible that only a limited number of subscribers to the FIND Twitter and LinkedIn accounts had relevant expertise in NCDs. Finally, the two-step design of the consensus building process allowed for a broad representation across countries and stakeholders; however, there was limited representation from certain high population middle-income countries such as China, and the geographical differences between the survey respondents and interviewees did not allow for confirmation of country-level feedback received at either stage.

## Conclusion

In conclusion, this TPP will inform developers and manufacturers considering the development of a cardiometabolic multi-parameter device for LMIC settings and will support decision makers to evaluate existing and future devices for their fit with TPP requirements.

## Supplementary Information

**Additional file 1**. Draft TPP (version 1). Draft TPP reviewed during semi-structured interviews and online survey.

**Additional file 2**. Semi-structured discussion guide. Discussion guide used in semi-structured interviews.

## Data Availability

All data generated or analysed during this study are included in this published article (and its Additional files 1, 2), with the exception of full survey results and interview feedback, which is withheld to protect the privacy of the participants.

## Abbreviations

CSLI: Clinical and Laboratory Standards Institute
CVD: Cardiovascular disease
HDL: High-density lipoprotein
LDL: Low-density lipoprotein
LMIC: Low- and middle income country
MSF: Médecins Sans Frontières
NCD: Non-communicable disease
PCR: Polymerase chain reaction
POC: Point of care
TC: Total cholesterol
TPP: Target product profile
USD: United States dollars
WHO: World Health Organization
