# Supplementary material for: Development of a target product profile for a point-of-care cardiometabolic device
Source: BMC Cardiovasc Disord. 2021 Oct 9;21:486. doi: 10.1186/s12872-021-02298-7 (PMC8501932; doi:10.1186/s12872-021-02298-7)
Supplement: Supplementary file 1 — Additional file 1. Draft TPP (version 1). Draft TPP reviewed during semi-structured interviews and online survey. [file 12872_2021_2298_MOESM1_ESM.docx]

**Supplementary File 1.** Draft TPP (version 1)

| **#** | **Characteristic** | **Min/Opt** | **Requirements** |
| --- | --- | --- | --- |
| **General** | | | |
| 1 | Intended use | Minimal | Intended for basic screening, diagnosis and management of cardiometabolic disorders (e.g. hyperlipidaemia, diabetes and renal function); excluding neonates |
| 1a |  | Optimal | Same as minimal, plus offering an expanded test menu to address a wider range of cardiometabolic disorders (e.g. liver function, acute cardiac care); including neonates |
| 2 | Description of the system | Minimal | Benchtop or hand-held instrument designed for use in combination with self-contained, disposable assay cartridge(s) or strips containing all required reagents to execute a test from sample to result |
| 2a |  | Optimal | Same as minimal |
| 3 | Target use setting | Minimal | Level 1 healthcare facility (primary care) defined as having a rudimentary equipped laboratory, water, electricity with intermittent surges and/or outages, limited climate control, dusty environment; medical staff onsite |
| 3a |  | Optimal | Level 0 healthcare facility without equipped laboratory, electricity with frequent surges and/or outages, no climate control, dusty environment; includes mobile testing facilities; medical staff onsite |
| 4 | Target user | Minimal | Minimally skilled healthcare worker (e.g. basic laboratory training, able to operate an integrated test system with minimal additional steps) |
| 4a |  | Optimal | Healthcare worker without specific laboratory training |
| **Device** | | | |
| 5 | Device design | Minimal | Device(s) with a single port capable of interfacing with one cartridge design or strip; one or more connectable instruments can be used to cover the minimal test menu (must be centrally managed) |
| 5a |  | Optimal | Single integrated device with universal port(s) capable of interfacing with one or more cartridge designs or strips for simultaneous, independent detection of multiple analytes |
| 6 | Size | Minimal | Small, table-top device (50x70x50 cm, or smaller) |
| 6a |  | Optimal | Hand-held |
| 7 | Weight | Minimal | ≤15 kg |
| 7a |  | Optimal | ≤1 kg |
| 8 | Power requirements | Minimal | Local 110–220 V AC mains power, plus uninterruptible power supply (UPS) to complete current cycle; UPS and circuit protector must be integrated within the system |
| 8a |  | Optimal | Same as minimal, with rechargeable battery back-up (8-hour operation) or single-use battery (for hand-held) |
| 9 | Throughput | Minimal | Throughput processing of one sample at a time; minimum of 10 samples per hour when individual analytes are tested or 4 samples per hour when analyte panels are tested |
| 9a |  | Optimal | More than one sample at a time with random access and the ability to test different analytes simultaneously |
| 10 | Environmental Stability: operating range of the device | Minimal | Operation at 10–35°C and up to 90% non-condensing humidity at an altitude up to 2,500 meters; able to function in direct sunlight; able to withstand dusty conditions |
| 10a |  | Optimal | Operation at 5–45°C and up to 98% non-condensing humidity at an altitude up to 3,000 meters; able to function in direct sunlight; able to withstand dusty conditions |
| 11 | Biosafety | Minimal | Closed, self-contained system with unprocessed sample transfer; no open handling of biohazardous material; easy decontamination of instrument surfaces |
| 11a |  | Optimal | Same as minimal |
| 12 | Training time needed | Minimal | Below 1 day for untrained healthcare worker |
| 12a |  | Optimal | Below 2 hours for untrained healthcare worker |
| 13 | Service, maintenance and calibration | Minimal | Daily maintenance (<30 minutes, with hands on time <10 minutes); mean time between failures of at least 24 months or 10,000 tests; self-check alerting operator to instrument errors or warnings; operator calibration per new lot or at set time intervals |
| 13a |  | Optimal | Weekly maintenance (<30 minutes, with hands on time <10 minutes); mean time between failures of at least 36 months or 30,000 tests; self-check alerting operator to instrument errors or warnings; ability to be calibrated remotely or no calibration needed (factory calibrated) |
| 14 | Patient identification capability | Minimal | Manual entry of alphanumeric patient identifier via keypad, touchscreen or connected result management device (e.g. smartphone) |
| 14a |  | Optimal | Same as minimal, plus bar code, radio frequency identification (RFID) or other reader |
| 15 | Result output | Minimal | Quantitative based on the analytes of detection; qualitative where this is sufficient to inform clinical decision making |
| 15a |  | Optimal | Quantitative plus option of qualitative readout where that result is sufficient to inform clinical decision-making; ability to select which test results are reported to the user based on the intended use in the regional context in which the test is used |
| 16 | Data display | Minimal | On-device visual readout with ability to function in various lighting conditions ranging from bright to low ambient light conditions; ability to add information (patient ID, operator ID, date, location, etc.) |
| 16a |  | Optimal | Same as minimal, with option to add custom result ranges and alerts to support clinical decision making |
| 17 | Connectivity | Minimal | Ability to connect to a mobile network, or Wifi or use a USB  for data transfer |
| 17a |  | Optimal | Same as minimal, including bluetooth  and bi-directional communication |
| 18 | Data export and protection | Minimal | Secured data export with end-to-end encryption connectivity to external printer; passcode-protected machine access |
| 18a |  | Optimal | Same as minimal, plus scheduled/automatic data export using interoperable standards; support of any or all of the following formats: HL7, FHIR, ASTM, JSON; passcode-protected individual user access |
| 19 | Memory | Minimal | 500 patient results, 100 quality control (QC) results |
| 19a |  | Optimal | 10,000 patient results, 20,000 QC results or unlimited data storage (cloud-based) |
| 20 | Manufacturing | Minimal | International Organization for Standardization (ISO) 13485:2016 compliant |
| 20a |  | Optimal | Same as minimal |
| 21 | List price of the device | Minimal | ≤5,000$ (USD) |
| 21a |  | Optimal | ≤300$ (USD) |
| 22 | Device regulatory status | Minimal | Approval through at least one Stringent Regulatory Authority (<http://www.stoptb.org/assets/documents/gdf/drugsupply/List_of_Countries_SRA.pdf>) |
| 22a |  | Optimal | Same as minimal plus CLIA-waived; WHO-PQ approval if requirements are in place |
| **Test cartridge/strip** | | | |
| 23 | Analytes/test menu | Minimal | Glucose, HbA1c, LDL (calculated from total cholesterol, HDL and triglycerides), creatinine |
| 23a |  | Optimal | Full lipid profile (values for cholesterol, HDL, LDL and triglycerides), liver enzymes (ALT, AST ALP GGT, Bilirubin*), troponin, BNP, ACR, auto calculation of eGFR and others as required for wider cardiometabolic disease management |
| 24 | Description of test cartridge/ strip | Minimal | Self-contained, disposable cartridge(s)/strips containing all required reagents, buffers or other consumables to execute a test from sample to result |
| 24a |  | Optimal | Same as minimal |
| 25 | Multiplexing of simultaneous tests | Minimal | Testing of one analyte at a time |
| 25a |  | Optimal | Testing of several analytes in parallel, either with multi-analyte panel cartridge, or with several cartridge/strip ports; ability to measure analytes individually, as well as part of a panel |
| 26 | Additional third party consumables | Minimal | None, except for sample collection |
| 26a |  | Optimal | None; manufacturer-provided kits contain all required items for sample collection and testing |
| 27 | Specimen type | Minimal | Ability to accept fingerstick whole blood |
| 27a |  | Optimal | Ability to accept fingerstick or venous whole blood, serum, plasma, urine |
| 28 | Sample volume | Minimal | Minimum sample volume required to reach clinically relevant sensitivities for each test but not more than 0.5 ml of sample (any type) per individual test or test panel |
| 28a |  | Optimal | Same as minimal but not more than 0.1 ml of sample (any type) per individual test or test panel |
| 29 | Limit of detection | Minimal | Equivalent to state of the art reference assays for the same target analytes; where applicable, clinically relevant LODs are to be met; for troponin, rule-out of myocardial infarction according to ACC/AHA guidelines |
| 29a |  | Optimal | Same as minimal; for troponin: rule-out of myocardial  infarction according to ESC 2018 guidelines |
| 30 | Interfering substances | Minimal | Level of interference established for haemolytic, lipaemic and icteric samples; level of interference established for exogenous and therapeutical substances |
| 30a |  | Optimal | Same as minimal |
| 31 | Standardization and traceability | Minimal | Test should be standardized based on established methods (e.g. isotope dilution mass spectrometry, ID-MS) and traceable to internationally recognised reference materials (where available) |
| 31a |  | Optimal | Same as minimal |
| 32 | Test result | Minimal | Quantitative result based on the analytes of detection. Qualitative result available to user where that result is sufficient to inform clinical decision making |
| 32a |  | Optimal | Same as minimal |
| 33 | Controls | Minimal | External positive and negative controls to be run with each new lot and every week |
| 33a |  | Optimal | External positive and negative controls to be run with each new lot and every month |
| 34 | Environmental stability: transport | Minimal | No cold chain required; should be able to tolerate stress during transport (cycles of temperature of 30 to 50°C) without affecting the labelled expiry date |
| 34a |  | Optimal | Same as minimal |
| 35 | Environmental Stability: Reagent shelf life | Minimal | 18 months at 2–35 °C (including 3 months at 40°C); 90% relative humidity |
| 35a |  | Optimal | 24 months at 2–40 °C; up to 98% relative humidity |
| 36 | Environmental Stability: Operating range | Minimal | 10–35°C; 90% relative humidity |
| 36a |  | Optimal | 5–45°C; 98% relative humidity |
| 37 | Waste/disposal Requirements | Minimal | No components that are classified with a GHS[1] classification – H(2) that would require waste disposal with high temperature incinerator (or more than a De Monfort type incinerator) |
| 37a |  | Optimal | Same as minimal |
| 38 | Manufacturing | Minimal | International Organization for Standardization (ISO) 13485:2016 compliant |
| 38a |  | Optimal | Same as minimal |
| 39 | Reagent regulatory status | Minimal | Approval through at least one Stringent Regulatory Authority (<http://www.stoptb.org/assets/documents/gdf/drugsupply/List_of_Countries_SRA.pdf> ) |
| 39a |  | Optimal | Same as minimal plus CLIA-waived; WHO-PQ approval if requirements are in place |
| 40 | List price of assay cartridge/  strips | Minimal | Strips: ≤1$ (USD); cartridges: ≤3$ (USD) per analyte (individual or as part of a panel) |
| 40a |  | Optimal | Strips: ≤0.5$ (USD); cartridges: ≤1$ (USD) per analyte (individual or as part of a panel) |
| 41 | Distribution territory | Minimal | Worldwide |
| 41a |  | Optimal | Same as minimal |

ACC, American College of Cardiology; ACR, albumin-to-creatinine ratio; AHA, American Heart Association; ALT, alanine aminotransferase; ALP, alkaline phosphatase; AST, aspartate aminotransferase; ASTM, American Society for Testing and Materials; BNP, brain natriuretic peptide; CLIA, Clinical laboratory improvement amendments; CSLI, Clinical and Laboratory Standards Institute; ESC, European Society of Cardiology; FHIR, fast healthcare interoperability resources; eGFR, estimated glomerular filtration rate; GGT, gamma-glutamyl transferase; GHS, globally harmonized system of classification and labelling of chemicals; HbA1c, glycated haemoglobin; HDL, high-density lipoprotein; HL7, health level 7; ID, identification; ID-MS, isotope dilution mass spectrometry; ISO, International Organization for Standardization; JSON, JavaScript object notation; LDL, low-density lipoprotein; LOD, limit of detection; RFID, radio frequency identification; QC, quality control; UPS, uninterruptible power supply; USB, Universal Serial Bus; USD, United States dollars; WHO-PQ, World Health Organization prequalification.
