# Supplementary material for: Development of a target product profile for a point-of-care cardiometabolic device
Source: BMC Cardiovasc Disord. 2021 Oct 9;21:486. doi: 10.1186/s12872-021-02298-7 (PMC8501932; doi:10.1186/s12872-021-02298-7)
Supplement: Supplementary file 2 — Additional file 2. Semi-structured discussion guide. Discussion guide used in semi-structured interviews. [file 12872_2021_2298_MOESM2_ESM.docx]

**Supplementary File 2.** Semi-structured discussion guide

| I have here a set of characteristics or attributes that are typically considered when it comes to POC cardiometabolic devices.   1. (MODERATOR EXPOSES/READS OUT LIST OF ATTRIBUTES) Please have a look at this and identify the TEN most important attributes   (NOTE: MODERATOR WILL EXPOSE EACH TPP CHARACTERISTIC AS TEXT IN A SCREENSHARED WHITEBOARD FACILITATED BY MODERATOR ON WEB-BASED PLATFORM. MODERATOR WILL ENSURE THE ORDER OF EXPOSURE WILL BE ROTATED PER RESPONDENT TO MANAGE ORDER BIAS. RESPONDENT WILL SEE/HEAR ONLY THE VALUES UNDER THE (TPPs) COLUMN. THE FOLLOWING TPP CHARACTERISTICS FROM THE DRAFT TPP (VERSION 1) WILL BE SHOWN, WITH MINIMAL AND OPTIMAL REQUIREMENTS. CHARACTERISTICS WILL BE EXPOSED ACCORDING TO RESPONDENT TYPE: U – ONLY Users, PDM – ONLY Purchase Decision Makers, UPDM – BOTH Users and Purchase Decision Makers)   - Intended use (UPDM) - Description of the system (UPDM) - Target use setting (UPDM) - Target user (UPDM) - Device design (UPDM) - Size (U) - Weight (U) - Power requirements (UPDM) - Throughput (PDM) - Environmental stability: operating range of the device (U) - Biosafety (UPDM) - Training time needed (UPDM) - Service, maintenance and calibration (U) - Patient identification capability (U) - Result output (U) - Data display (U) - Connectivity (UPDM) - List price of the device (PDM) - Analytes/test menu (U) - Description of test cartridge/strip (U) - Multiplexing of simultaneous tests (UPDM) - Additional third party consumables (PDM) - Specimen type (U) - Test result (U) - Controls (UPDM) - Environmental stability: transport (PDM) - Environmental stability: reagent shelf life (UPDM) - Environmental stability: operating range (UPDM) - List price of assay cartridge/strips (PDM)  1. Looking at these 10 that you have selected, now please rank them from 1-10, starting with 1 being the most important. 2. For each rank that you have provided – please suggest what should be the most optimal and minimal level of capability for each characteristic. (Moderators to probe on TPP phrasing). |
| --- |
